# Mechanistic Study of the Spiroindolones: A New Class of Antimalarials

**DOI:** 10.3390/molecules170910131

**Published:** 2012-08-24

**Authors:** Bin Zou, Peiling Yap, Louis-Sebastian Sonntag, Seh Yong Leong, Bryan K. S. Yeung, Thomas H. Keller

**Affiliations:** 1 Novartis Institute for Tropical Diseases, 10 Biopolis Road, #05-01 Chromos, Singapore; 2 Experimental Therapeutics Centre, 31 Biopolis Drive, #03-01 Nanos, Singapore; Email: thkeller@etc.a-star.edu.sg

**Keywords:** malaria, spiroindolones, NITD609, Pictet-Spengler reaction, mechanism

## Abstract

During the synthesis of the new antimalarial drug candidate NITD609, a high degree of diastereoselectivity was observed in the Pictet-Spengler reaction. By isolating both the **4*E*** and **4*Z*** imine intermediates, a systematic mechanistic study of the reaction under both kinetic and thermodynamic conditions was conducted. This study provides insight into the source of the diastereoselectivity for this important class of compounds.

## 1. Introduction

Malaria continues to be a significant global health problem, with an estimated 216 million infections and 655,000 deaths in 2010 alone [1]. In light of increasing resistance to many current antimalarials, and the growing concern over reduced effectiveness of artemisinin-combination therapies in the long term, there is an urgent need for new drug candidates with the potential to replace the artemisinins in the treatment of malaria [2,3,4].

**Figure 1:**
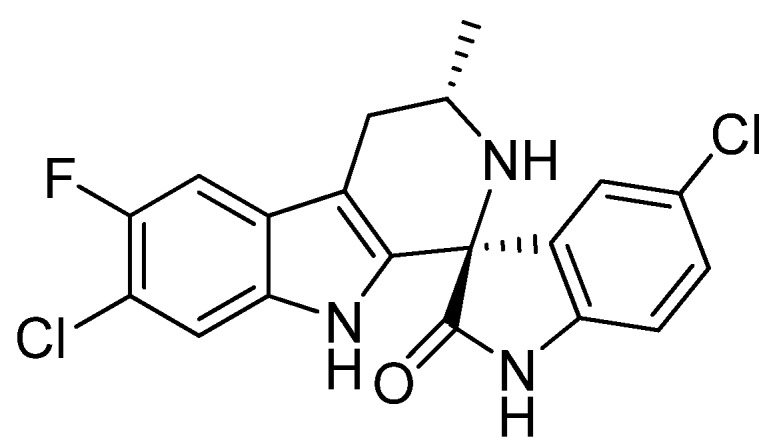
Structure of NITD609.

We recently reported the new antimalarial drug candidate NITD609 (Figure 1), which exhibits excellent oral bioavailability and exceptional efficacy in a rodent malarial model [5]. While the initial discovery of this new chemotype with potent antimalarial activity was welcome, the presence of the quaternary center in the structure was a cause for some concern, since it was unclear whether the relative stereochemistry of the two chiral centers could be adequately controlled. This issue became especially critical when it was determined that only a single diastereoisomer exhibited the desired level of antimalarial activity [6].

**Scheme 1:**
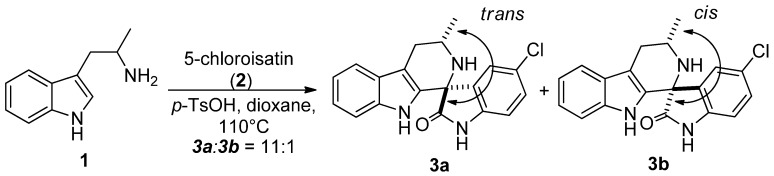
Diastereoselectivity in the Pictet-Spengler reaction of spiroindolones.

The synthesis of NITD609 and its analogues features a highly diastereoselective Pictet-Spengler reaction. In the reaction of *rac*-α-methyltryptamine (**1**) [6] with 5-chloroisatin (**2**) (Scheme 1) the formation of the *trans* diastereoisomer **3a** (where the methyl and the carbonyl groups are in a relative *trans* configuration) was favoured. Although this result was encouraging as the major diastereoisomer **3a** was found to contain the most active stereoisomer required for antimalarial activity [6], the source of the stereoselectivity was unclear. In this paper, a mechanistic study of this reaction is reported.

## 2. Results and Discussion

The asymmetric Pictet-Spengler reaction has been widely reported [7,8,9,10,11,12,13], and recent literatures have demonstrated very encouraging progress on enantioselective synthesis of this spiroindolone class of compounds using chiral acids as the catalyst [14,15]. However, high diastereoselectivity is not often seen in reactions with α-methyltryptamines [16,17,18]. Although the mechanism for the diastereoselective Pictet-Spengler reaction has been studied by a number of groups [19,20,21,22,23,24], the influence of the imine geometry on the diastereoselectivity has received little attention [25]. Since the importance of imine geometry has been discussed in other types of reactions [26,27,28], we were tying to investigate the diastereoselectivity source starting from the imine intermediates. An important aspect of our work compared to previous investigations is that the relatively lower reactivity of the imines derived from isatins allowed for the isolation and characterization of imine intermediates, which provided additional insights into the drivers of diastereoselectivity.

Abadi and coworkers reported the synthesis of a series of isatin-derived imines as kinase inhibitors [29]. By adapting the Abadi conditions to our system, we were able to isolate the desired imines in acceptable yield by reacting **1** and **2** in refluxing ethanol. Moreover optimization of the reaction conditions provided access to both imines **4*E*** (as a 23:1 mixture of imines) and **4*Z*** (1:20) separately (Table 1, entries 1 and 2). The relative stability of the imines allowed us to isolate and assign the configuration of the isomers by NOESY-1D NMR spectroscopy (see Supporting Information) [30].

**Table 1:** Preparation of imines intermediates. 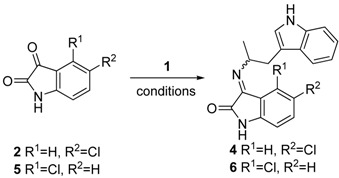

Entry	Isatin	Conditions *^a^*	Yield	Product
1	2	2.85 M, EtOH, 80 °C	58%	**4*E*:4*Z*** (23:1) *^b^*
2	2	0.95 M, EtOH, 80 °C	58%	**4*E*:4*Z*** (1:20) *^b^*
3	5	0.95 M, EtOH, 80 °C	55%	**6*Z^c^***

*^a^* reaction concentration, solvent, reaction temperature; *^b^* ratio of configuration isomers determined by ^1^H-NMR; *^c^* **6*Z*** was the sole product observed in ^1^H-NMR.

**Scheme 2:**
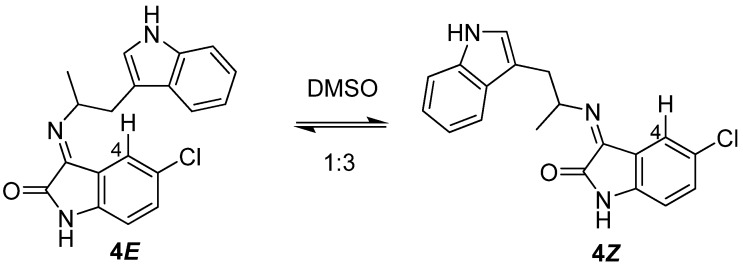
Thermodynamic mixture of imines **4**.

Access to both *E* and *Z* imine products in virtually pure form was rather fortuitous and seemed to be facilitated by the different physical properties of the two imines. This phenomenon was not studied in detail, however both the ***4E*** and ***4Z*** isomers can be reproducibly prepared by varying the reaction concentration as described in Table 1. Both imines isomerized over 24 h in DMSO at room temperature to a 1:3 mixture of **4*E***:**4*Z*** (Scheme 2). The preference for the *Z* imine in the thermodynamic mixture can be rationalized by the unfavorable steric interaction between the H4 of the isatin moiety and α-methyltryptamine. Indeed increasing the steric bulk at the 4-position as in 4-chloroisatin (**5**) only produced **6*Z*** (Table 1, entry 3), which did not isomerize under the described conditions.

With the individual *E* and *Z* isomers in hand, we turned our attention to the cyclization reaction. Similar diastereoselectivities could be achieved when ethyl acetate was used as the solvent in the reaction shown in Scheme 1, therefore, ethyl acetate was chosen as the solvent since it allowed us to run reactions at low temperature.

When **4*Z*** was cyclized in the presence of HCl (4 N in 1,4-dioxane, 10 eq.) at three different temperatures, an excellent yield of the corresponding tetrahydro-*β*-carboline was obtained (Table 2, entry 1). In all instances *trans* isomer **3a** was the major product, irrespective of the reaction conditions, although there was a clear trend towards higher diastereoselectivity at lower temperatures. In contrast the cyclization of **4*E*** at −78 °C provided the *cis* product **3b** preferentially with excellent diastereoselectivity, while higher reaction temperatures led to a reversal of the diastereoselectivity until *trans* product **3a** predominated at 110 °C (Table 2, entry 2). The same trend was observed when the thermodynamic mixture of imines was cyclized (Table 2, entry 3). At low temperature the product ratio was similar to the starting ratio of the imines, while at high temperature the *trans* product predominated. Finally when **6*Z*** was cyclized, the results were analogous to **4*Z*** (Table 2, entry 4), the reaction at −78 °C providing *trans* product **7a**, but this time with exquisite stereoselectivity, while at 110 °C the selectivity was reduced to 12:1.

**Table 2:** Diastereoselectivity of the Pictet-Spengler reaction. 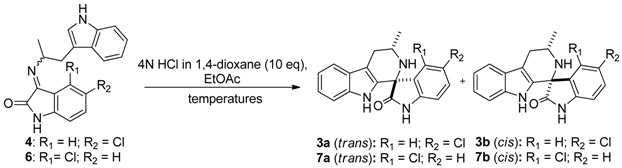

Entry	Imine	Product	*trans:cis* ratio *^a^* (*yield %*) *^b^*		
			−78 °C *^c^*	r.t. *^d^*	110 °C *^e^*
1	**4*E*:4*Z*** (1:20)	**3a/3b**	18:1(*100*)	12:1(*100*)	10:1(*83*)
2	**4*E*:4*Z*** (23:1)	**3a/3b**	1:20(*95*)	1:2(*100*)	7:1(*97*)
3	**4*E*:4*Z*** (1:3)	**3a/3b**	1:1(*100*)	3:1(*94*)	11:1(*100*)
4	**6*Z***	**7a/7b**	165:1(*93*)	37:1(*98*)	12:1(*92*)

*^a^* ratios determined by HPLC; *^b^* isolated yields for *trans* and *cis* mixture; *^c ^*reaction time, 1 h; *^d^* room temperature; reaction time; 25 min; *^e^* reaction time, 10 min.

**Scheme 3:**
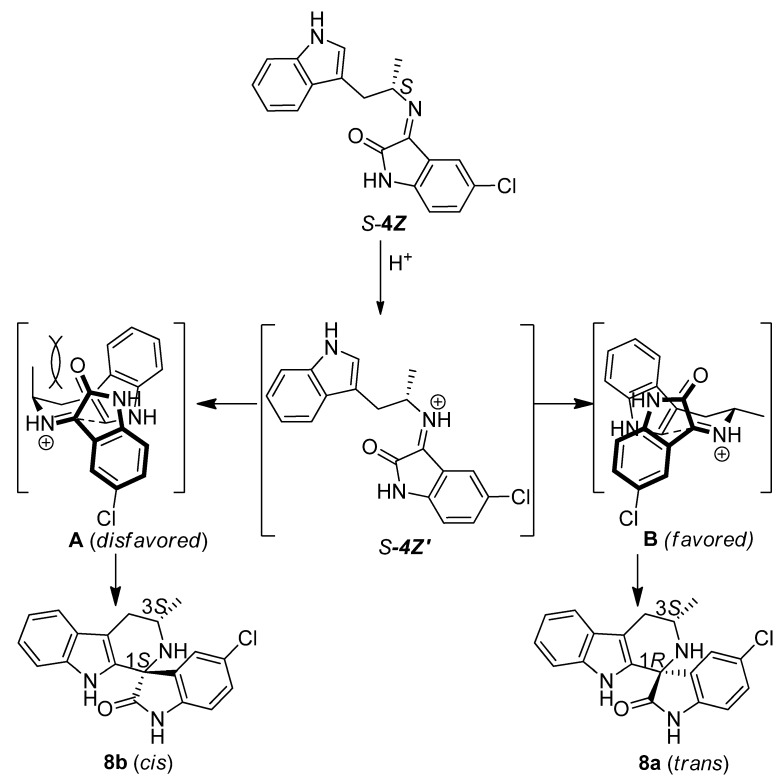
Proposed mechanism for the cyclization of the *S*-**4*Z*** imine under kinetic conditions favoring *trans* product.

The ability to isolate the imine intermediates allowed us to gain detailed insight into the kinetic control mechanism. The results in Table 2 clearly show that the imine configuration is the major determinant for the diastereoselectivity at −78 °C. In all cases of kinetic control, the mixture of *cis* and *trans* spirotetrahydro-*β*-carbolines is basically equivalent to the original ratio of *E* and *Z* imines. Based on these results we propose a chair-like transition state for the cyclization step of the Pictet-Spengler reaction (Scheme 3).

The *Z*imine *S-***4*Z*** is first of all protonated under acidic conditions to generated intermediate *S*-***4Z′***, which can assume two possible conformations, leading to two diastereomeric transition states **A** and **B**, which differ in their face of attack on the imine (Scheme 3). Depending on the conformation of the six-membered transition state the methyl group is either in a pseudoaxial (**A**) or in a pseudoequatorial (**B**) position. The pseudoaxial transition state **A**, is less favored, as it leads to A^1,3^ strain between the methyl group and the carbonyl of the isatin. Thus, the pseudoequatorial transition state **B** is favored, which results in the formation of the *trans* product **8a** (a similar mechanism for the cyclization of the *R-***4*Z*** enantiomer equally favors the *trans* product). A similar mechanism for the cyclization of *E* imine affords *cis* product **8b** as the major isomer.

**Scheme 4:**
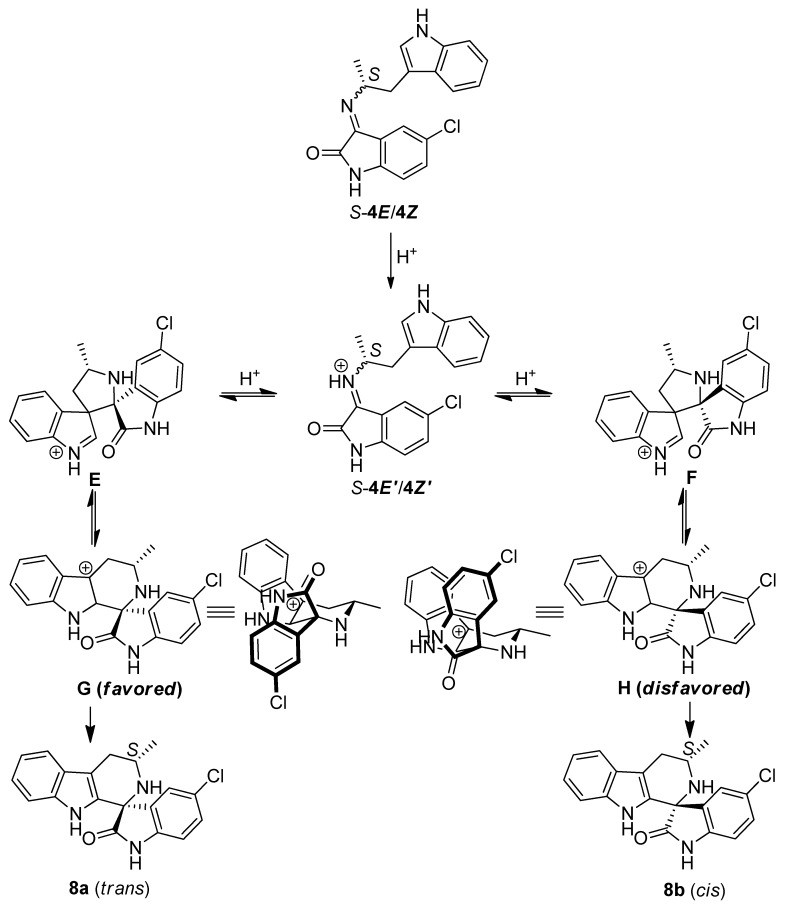
Proposed mechanism for the cyclization of the *S*-imine under thermodynamic conditions favoring *trans* product.

Our findings show that under thermodynamic conditions, the *E:Z* ratio of the imine does not influence the diastereoselectivity. Isomerization of the imine at high temperatures cannot explain the results in Table 2, as imine **6*Z***, which due to steric hindrance cannot adopt the *E* configuration, shows similar diastereoselectivity as imines **4*E*** and **4*Z***. These observations suggest that a different mechanism is responsible for the product distribution. One possible explanation invokes a mechanism similar to the one proposed by Bailey and co-workers [19,20,21]. Under thermodynamic conditions, bis-spiro intermediates, **E** and/or **F**, are formed independent of the imine geometry (**4*E*** or **4*Z***, Scheme 4). The formation of the bis-spiro intermediates is fast and reversible and hence will not influence the stereochemistry of the final products [20]. Instead, bond migration to form the central six-membered ring in intermediates **G** and **H**, is rate determining [20]. The two diastereomeric cations **G** and **H** differ only in the configuration of the spirocenter, this leads to either the lactam (**G**) or the chloro-phenyl portion (**H**) of the isatin to occupy the pseudo-axial position. Of these two possibilities, intermediate **G** is favored due to its lower A^1,3^ strain, leading to **8a** to be the major product formed.

**Scheme 5:**
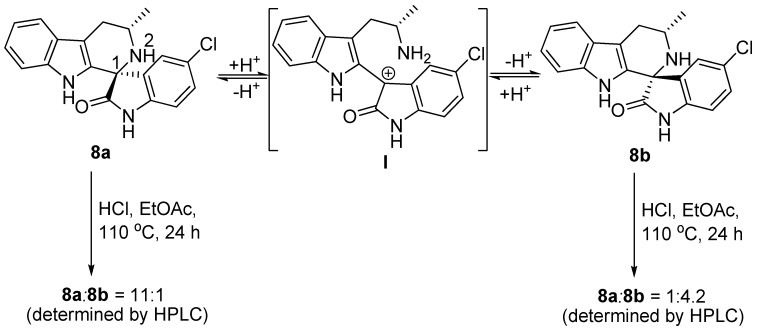
A proposed mechanism of isomerization between **8a** and **8b** under acidic conditions.

The results observed under thermodynamic control in the Pictet-Spengler reaction could also be explained by the acid catalyzed scission of the C1-N2 bond of the spirocenter (Scheme 5) [8,23,24]. In order to determine whether this isomerization could explain the observed diastereoselectivities, we subjected both the pure **8a** and **8b** isomers to our standard reaction conditions at 110 °C for extended reaction times (24 h). Although a slight epimerisation of the spirocenter was observed (Scheme 5), essentially the starting materials remained unchanged. These results suggest that this isomerization is not fast enough to explain the results in Table 2. This is not surprising, since the formation of a carbocation at C1 leads to a disfavored intermediate (**I**).

## 3. Experimental

### 3.1. Materials and Reagents

Reagents and solvents were purchased from Aldrich, Acros, or other commercial sources and used without further purification. Thin layer chromatography (TLC) was performed on precoated silica gel 60 F_254_ plates from Merck. Compounds were visualized under UV light, ninhydrin, or phosphomolybdic acid (PMA) stain. NMR spectra were obtained on a Varian 300 MHz Mercury NMR using CDCl_3_, and DMSO-*d_6_* as solvents. Compound purity was determined by LC/MS and HPLC and carried out on an Agilent LC110 HPLC equipped with a Waters Symmetry Shield RP18, 3.5 μm, 4.6 × 150 mm column using a gradient (13 min) of 95:5 H_2_O (0.1% formic acid):CH_3_CN to 5:95 H_2_O (0.1% formic acid):CH_3_CN. The purity of all compounds reported were >95% measured at 254 nm. The melting point was measured with BÜCHI B-540.

### 3.2. Synthesis of Imines 4Z, 4E, and 6Z

*(Z)-3-[(2-1H-Indole-4-yl)isopropylimino]-5-chloroindolin-2-one* (**4*Z***). Methyltryptamine (**1**, 100.0 mg, 0.57 mmol) and 5-chloroisatin (**2**, 104.2 mg, 0.57 mmol) were dissolved in dry ethanol (0.6 mL) in a sealed tube. The resulting clear orange red solution was stirred and heated at 80 °C for 1.5 h. A yellow precipitate was observed after 40 min of stirring. After completion of the reaction, the precipitate was collected via filtration, washed with cold ethanol and dried under vacuum. The title compound was isolated as a yellow powder (113.0 mg, 58% yield). **4*Z***: m.p. 181.3–182.0 °C; IR (film): *ν*_max_ = 1707 cm^−1^; ^1^H-NMR (DMSO-*d*_6_): δ = 10.98 (br.s., 1H), 10.76 (s, 1H), 7.63 (d, *J* = 7.5 Hz, 1H), 7.41–7.46 (m, 1H), 7.40 (d, *J* = 2.1 Hz, 1H), 7.30 (d, *J* = 7.8 Hz, 1H), 7.10 (d, *J* = 2.1 Hz, 1H), 7.03 (ddd, *J* = 8.1, 7.2, 1.2 Hz, 1H), 6.93 (ddd, *J* = 8.1, 7.2, 1.2 Hz, 1H), 6.84 (dd, *J* = 8.1, 1.2 Hz, 1H), 5.56–5.66 (m, 1H), 2.88–3.01 (m, 2H),1.19 ppm (d, *J* = 6.0 Hz, 3H); ^13^C-NMR (DMSO-*d*_6_): δ = 160.2, 150.1, 140.3, 135.7, 131.8, 128.3, 127.5, 123.4, 122.2, 122.1, 119.0, 118.9, 113.0, 112.9, 110.6, 110.5, 56.2, 33.8, 21.3 ppm.

*(E)-3-[(2-1H-Indole-4-yl)isopropylimino]-5-chloroindolin-2-one* (**4*E***). Methyltryptamine (**1**, 100.0 mg, 0.57 mmol) and 5-chloroisatin (**2**, 104.2 mg, 0.57 mmol) were dissolved in dry ethanol (0.2 mL) in a sealed tube. The resulting clear orange red solution was stirred and heated at 80 °C for 1 h. A yellow precipitate was observed after 5 min of stirring. After completion of the reaction, the precipitate was collected via filtration, washed with cold ethanol and dried under vacuum. The title compound was isolated as a bright yellow powder (113.0 mg, 58% yield). **4*E***: m.p. 168.0–169.2 °C; IR (film): *ν*_max_ = 1728 cm^−1^; ^1^H-NMR (DMSO-*d*_6_): δ = 10.93 (br.s., 1H), 10.77 (s, 1H), 7.59 (d, *J* = 8.1 Hz, 1H), 7.53 (d, *J* = 2.1 Hz, 1H), 7.37 (dd, *J* = 8.4, 2.1 Hz, 1H), 7.28 (ddd, *J* = 7.8, 1.2, 1.2 Hz, 1H), 7.10 (d, *J* = 2.1 Hz, 1H), 7.03 (td, *J* = 7.5, 1.2 Hz, 1H), 6.96 (ddd, *J* = 7.8, 6.6, 1.2 Hz, 1H), 6.85 (d, *J* = 8.4 Hz, 1H), 4.57–4.69 (m, 1H), 2.97–3.14 (m, 2H), 1.33 ppm (d, *J* = 6.2 Hz, 3H); ^13^C-NMR (DMSO-*d*_6_): δ = 158.2, 151.0, 142.3, 135.6, 131.9, 127.7, 127.3, 126.0, 122.2, 119.2, 118.3, 117.2, 112.9, 112.4, 111.8, 110.5, 58.9, 33.6, 20.6 ppm.

*(Z)-3-[(2-1H-Indole-4-yl)isopropylimino]-4-chloroindolin-2-one* (**6*Z***). Methyltryptamine (**1**, 100.0 mg, 0.57 mmol) and 4-chloroisatin (**5**, 104.2 mg, 0.57 mmol) were dissolved in dry ethanol (0.6 mL) in a sealed tube. The resulting clear orange solution was stirred and heated at 80 °C for 2.5 h. After completion of the reaction, the precipitate was collected via filtration, washed with cold ethanol and dried under vacuum. The title compound was isolated as a yellow powder (107.3 mg, 0.32 mmol, 55% yield). **6*Z***: m.p. 169.4–170.3 °C; IR (film): *ν*_max _=1708 cm^−1^; ^1^H-NMR (DMSO-*d*_6_): δ = 11.05 (br.s., 1H), 10.76 (s, 1H), 7.66 (d, *J* = 7.5 Hz, 1H), 7.27–7.38 (m, 2H), 7.13 (d, *J* = 2.1 Hz, 1H), 6.99–7.06 (m, 2H), 6.93 (ddd, *J* = 7.8, 7.2, 1.2 Hz, 1H), 6.78 (dd, *J* = 7.8, 1.2 Hz, 1H), 5.63–5.73 (m, 1H), 2.89–3.04 (m, 2H), 1.20 ppm (d, *J* = 6.0 Hz, 3H); ^13^C-NMR (DMSO-*d*_6_): δ = 158.5, 151.0, 145.7, 136.1, 133.4, 128.9, 127.5, 123.8, 123.5, 120.7, 118.7, 118.1, 117.4, 111.7, 111.2, 109.2, 55.8, 34.0, 21.7 ppm.

### 3.3. General Procedure for Cyclization of Imines at Different Temperatures

To the solution of the imines (20 mg, 0.06 mmol) in ethyl acetate (1 mL) was added hydrochloric acid (0.15 mL, 4 N in 1,4-dioxane, 10.0 eq.) at −78 °C, room temperature or 110 °C (in sealed tube) and the reaction mixture was stirred for 1 h, 25 min or 10 min respectively. The reaction mixture was quneched by adding 1 N aqueous sodium hydroxide solution (3 mL) and aqueous phase was extracted with ethyl acetate (2 × 8 mL). The combined organic phases were dried over Na_2_SO_4_, filtered, and concentrated in *vacuo*. The residue was purified by flash column chromatography.

*(trans)-5'-Chloro-3-methyl-2,3,4,9-tetrahydrospiro[β-carboline-1,3'-indol]-2'(1'H)-one* (**3a**): *m/z* (ESI): [M+H]^+^ 338; ^1^H-NMR (DMSO-*d_6_*): δ = 10.45 (s, 1H), 10.42 (s, 1H), 7.43 (d, *J* = 7.2 Hz, 1H), 7.31 (dd, *J* = 8.4, 2.4 Hz, 1H), 7.16 (d, *J* = 7.2 Hz, 1H), 7.03 (d, *J* = 2.4 Hz, 1H), 6.99 (m, 1H), 6.92 (d, *J* = 8.4 Hz, 2H), 3.93 (m, 1H), 3.05 (d, *J* = 6.3 Hz, 1H), 2.79 (dd, *J* = 15.0, 3.6 Hz, 1H), 2.41 (dd, *J* = 15.0, 10.5 Hz, 1H), 1.17 ppm (d, *J* = 6.3 Hz, 3H); ^13^C-NMR (DMSO-*d*_6_): δ = 178.5, 141.6, 136.4, 134.4, 131.0, 128.9, 126.4, 125.5, 124.8, 121.1, 118.4, 117.8, 111.1, 111.0, 61.9, 44.3, 29.6, 21.7 ppm.

*(cis)-5'-Chloro-3-methyl-2,3,4,9-tetrahydrospiro[β-carboline-1,3'-indol]-2'(1'H)-one* (**3b**): *m/z* (ESI): [M+H]^+^ 338; ^1^H-NMR (DMSO-*d_6_*): δ = 10.80 (s, 1H), 10.59 (s, 1H), 7.45 (d, *J* = 6.9 Hz, 1H), 7.30 (dd, *J* = 8.4, 2.1 Hz, 1H), 7.18(d, *J* = 2.1 Hz, 1H), 7.15 (s, 1H), 7.03 (td, *J* = 7.5, 1.5 Hz, 1H), 6.94–7.00 (m, 2H), 3.47 (m, 1H), 2.92 (dd, *J* =15.3, 3.9 Hz, 1H), 2.42 (dd, *J* = 15.3, 10.5 Hz, 1H), 2.31 (d, *J* = 9.0 Hz, 1H), 1.23 ppm (d, *J* = 6.3 Hz, 3H); ^13^C-NMR (DMSO-*d*_6_): δ = 177.1, 140.6, 136.4, 135.9, 130.7, 128.5, 126.1, 125.7, 124.3, 121.3, 118.4, 117.8, 111.6, 111.1, 110.8, 62.9, 45.8, 29.7, 22.0 ppm.

*(trans)-4'-Chloro-3-methyl-2,3,4,9-tetrahydrospiro[β-carboline-1,3'-indol]-2'(1'H)-one* (**7a**): *m/z* (ESI): [M+H]^+^ 338; ^1^H-NMR (DMSO-*d*_6_): δ = 10.55 (br.s., 1H), 10.50 (s, 1H), 7.43 (d, *J* = 7.2 Hz, 1H), 7.29 (t, *J* = 7.8 Hz, 1H), 7.17 (d, *J* = 7.2 Hz, 1H), 6.94–7.05 (m, 2H), 6.85–6.94 (m, 2H), 3.94 (m, 1H), 2.84 (dd, *J* = 15.0, 3.6 Hz, 1H), 2.65 (d, *J* = 6.0 Hz, 1H), 2.36 (dd, *J* = 15.0, 10.5 Hz, 1H), 1.19 ppm (d, *J* = 6.6 Hz, 3H); ^13^C-NMR (DMSO-*d*_6_): δ = 177.5, 144.8, 136.4, 130.9, 130.5, 129.4, 128.2, 126.3, 122.4, 120.9, 118.2, 117.7, 111.3, 111.1, 108.7, 62.3, 44.3, 29.7, 21.8 ppm.

*(cis)-4'-Chloro-3-methyl-2,3,4,9-tetrahydrospiro[β-carboline-1,3'-indol]-2'(1'H)-one* (**7b**): *m/z* (ESI): [M+H]^+^ 338; ^1^H-NMR (DMSO-*d*_6_): δ = 10.83 (br.s., 1H), 10.50 (s, 1H), 7.44 (d, *J* = 7.2 Hz, 1H), 7.28 (d, *J* = 8.1 Hz, 1H), 7.17 (d, *J* = 7.5 Hz, 1H), 6.90–7.06 (m, 4H), 3.80 (m, 1H), 2.92 (dd, *J* = 15.0, 3.9 Hz, 1H), 2.41 (dd, *J* = 15.3, 10.2 Hz, 1H), 1.23 ppm (d, *J* = 6.0 Hz, 3H); ^ 13^C-NMR (DMSO-*d*_6_): δ = 177.3, 144.4, 136.4, 130.5, 130.5, 130.1, 129.1, 126.2, 122.9, 121.1, 118.2, 117.8, 111.3, 111.1, 109.2, 63.6, 46.9, 29.9, 22.2 ppm.

## 4. Conclusions

In summary, an explanation of the high diastereoselectivity observed in the Pictet-Spengler reaction of the new class of antimalarials exemplified by the candidate NITD609 is proposed. A careful mechanistic study of the reaction, including the isolation and characterization of the imine intermediates, suggests that under kinetic conditions, the geometry of the imine and subsequent release of A^1,3^ strain in the six-membered transition state determines the *cis*/*trans* ratio and thus the diastereoselectivity of the products; under thermodynamic conditions, the diastereoselective outcome is independent of the imine geometry and instead controlled by fast equilibration through a bis-spiro intermediate. The subsequent ring expansion forms a six-membered transition state which is governed by the release of A^1,3^ strain. The lower energy intermediate leads to the formation of the favored *trans* product. This knowledge has proven helpful in the large-scale synthesis of our clinical candidate NITD609.

## Supplementary Materials

Supplementary materials can be accessed at: http://www.mdpi.com/1420-3049/17/9/10131/s1.
