# Supplementary figures and images for: Mechanistic Study of the Spiroindolones: A New Class of Antimalarials
Source: Molecules. 2012 Aug 24;17(9):10131–41. doi: 10.3390/molecules170910131 (PMC6268731; doi:10.3390/molecules170910131)

# Supplementary Materials

## NOESY 1D-NMR for 4Z and 4E

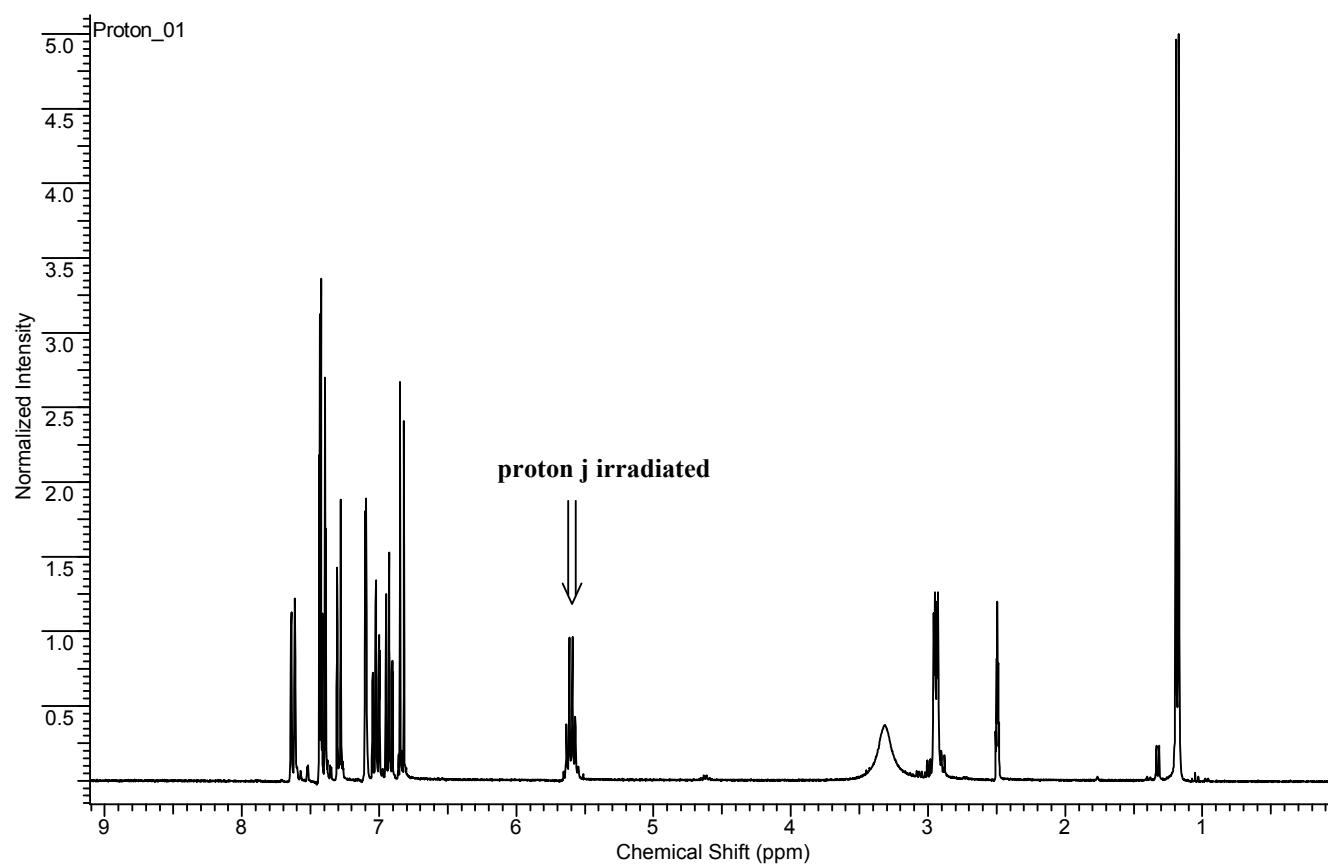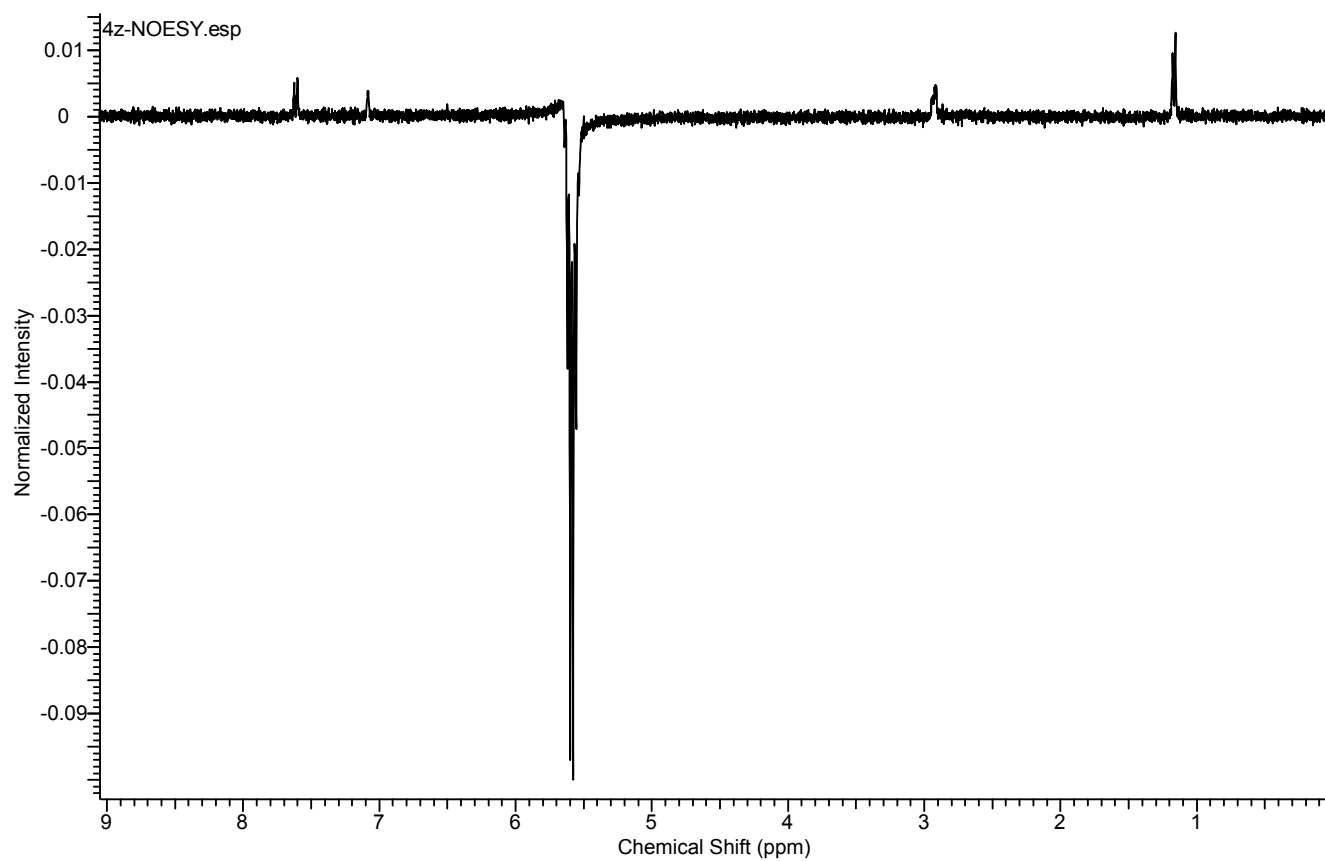

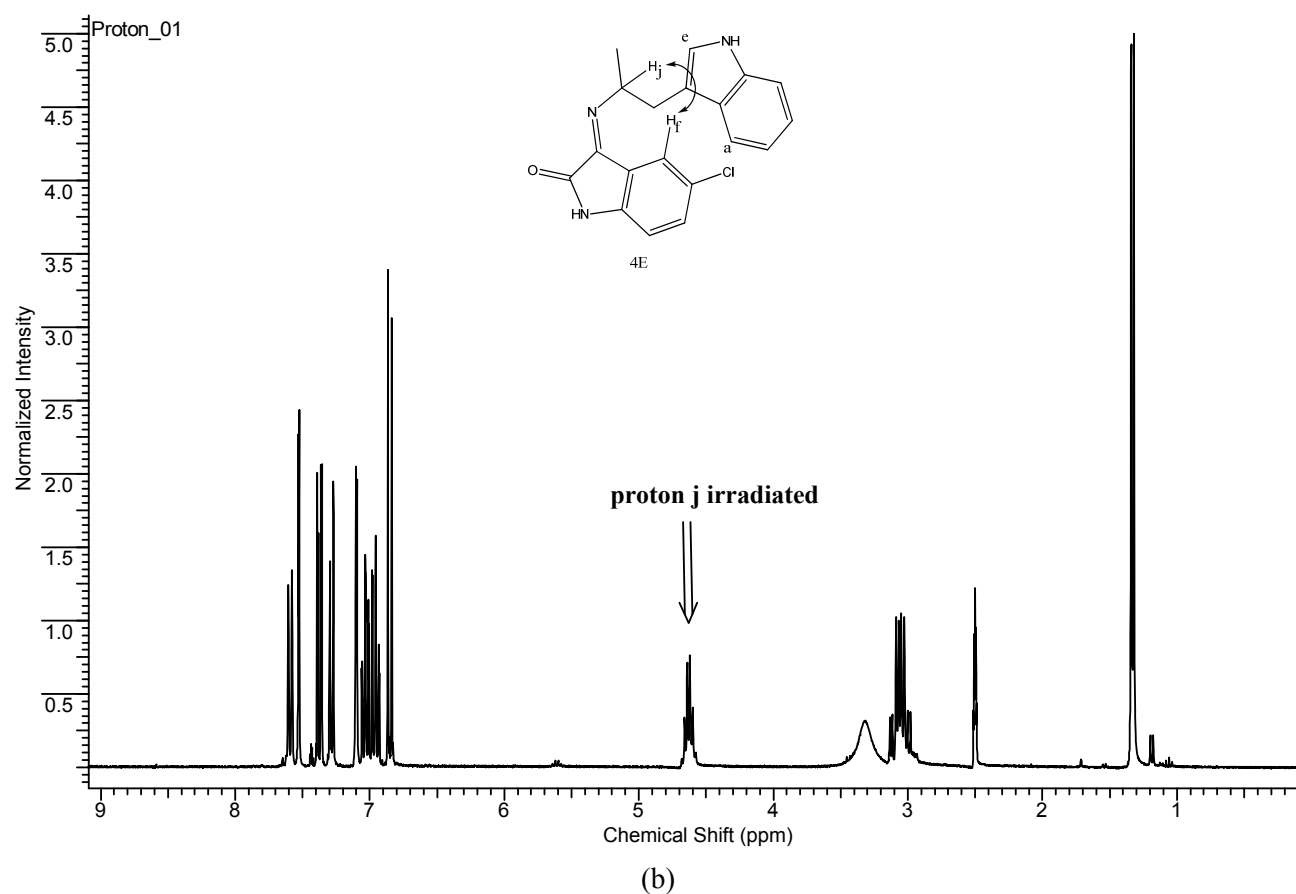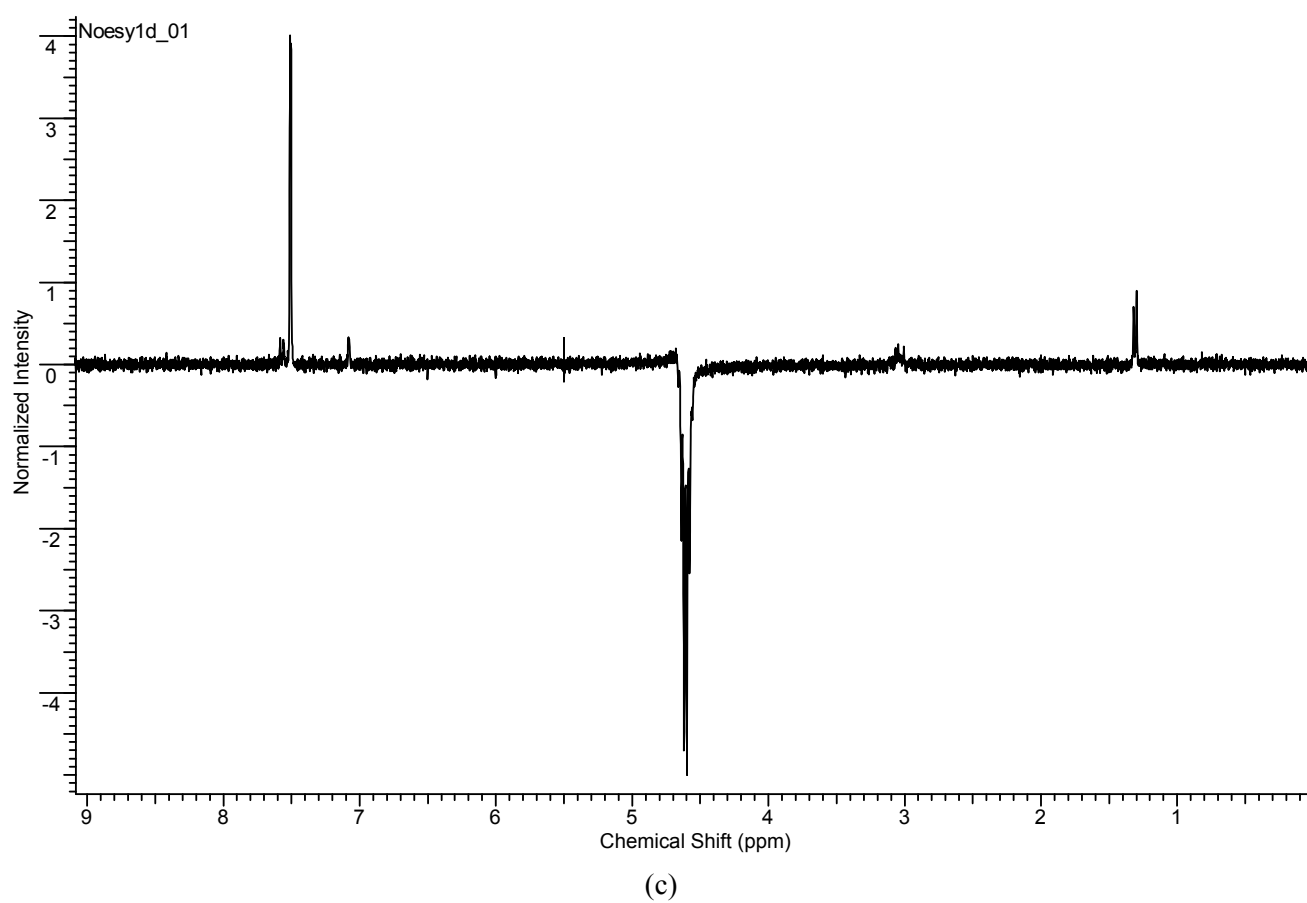

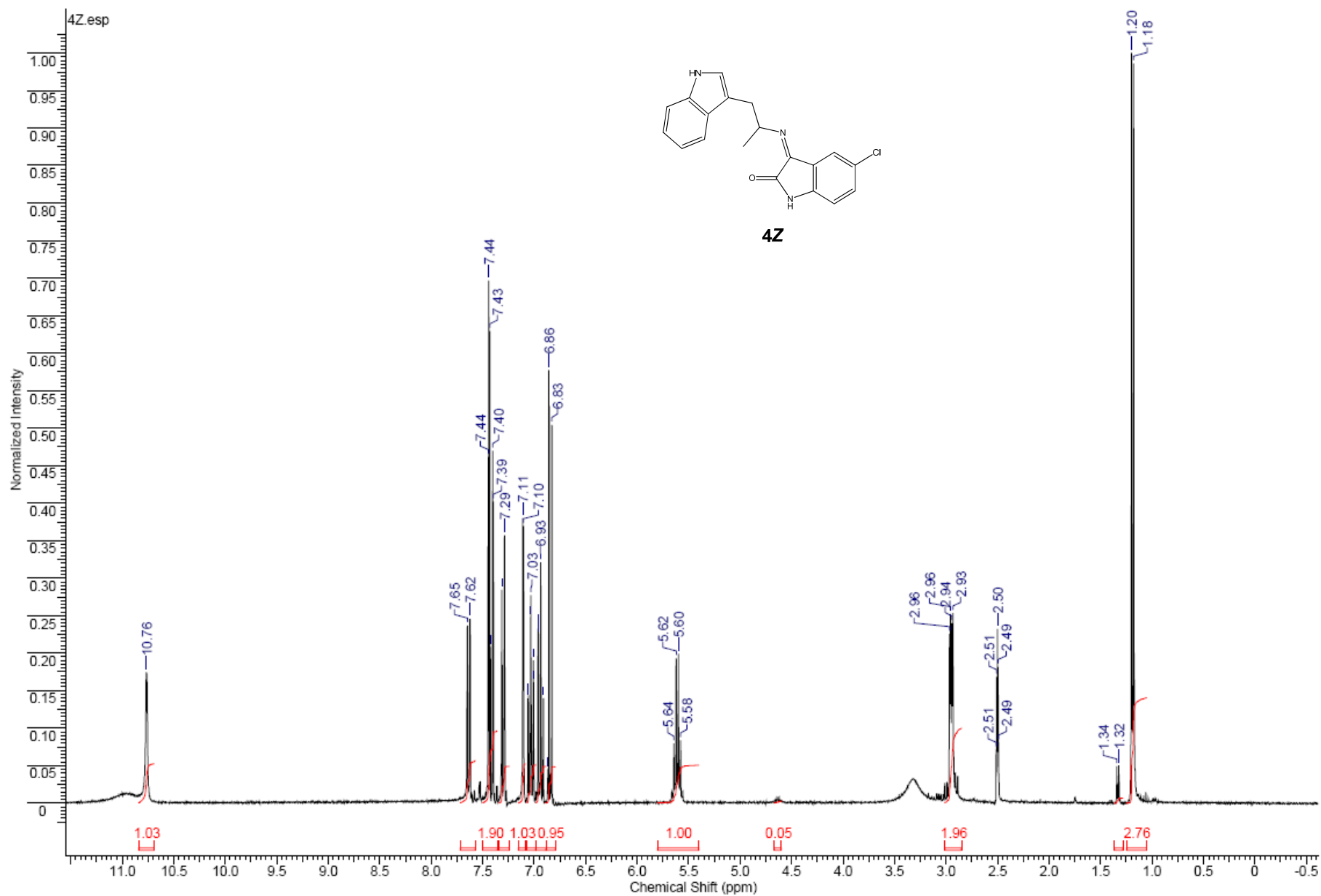

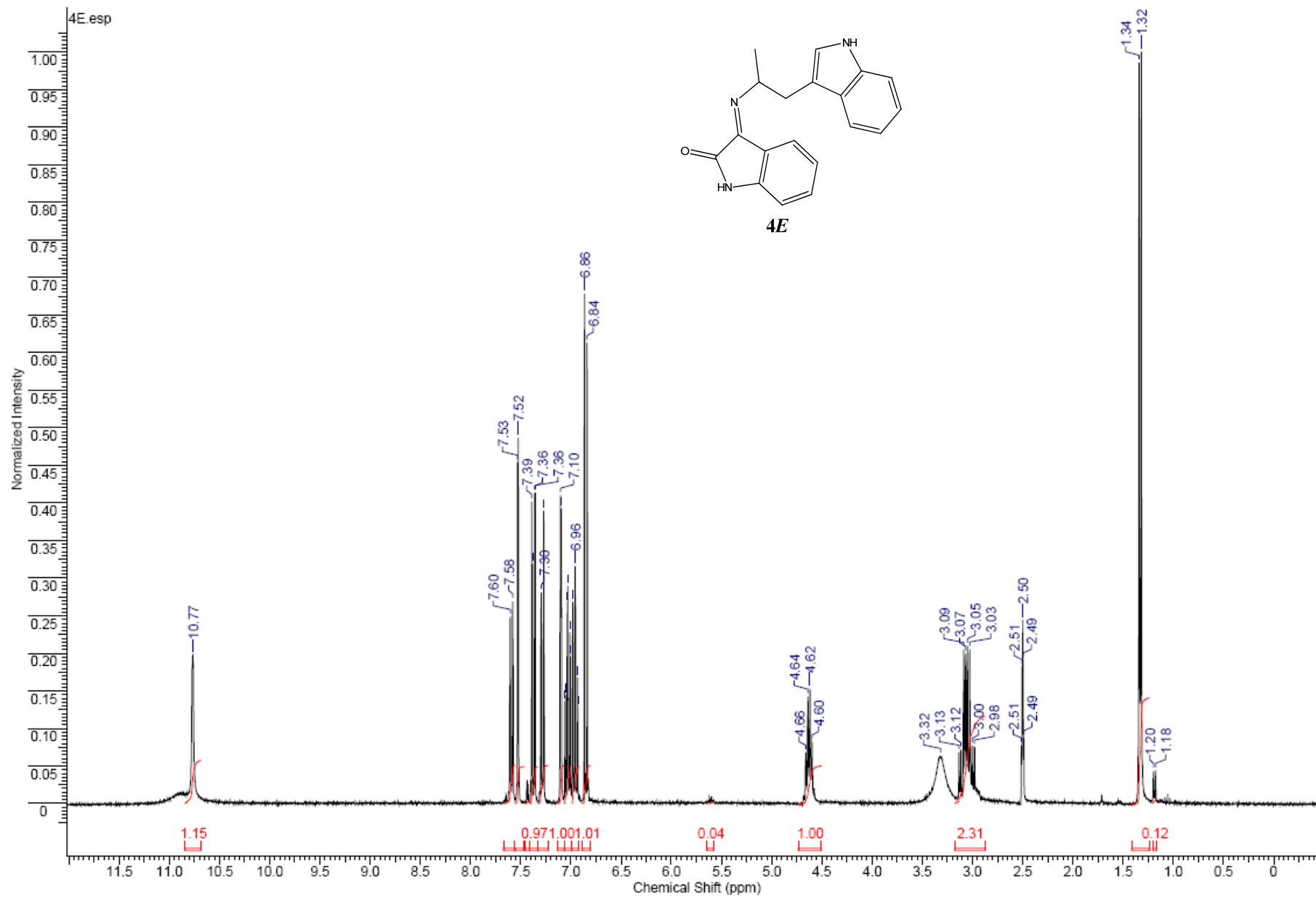

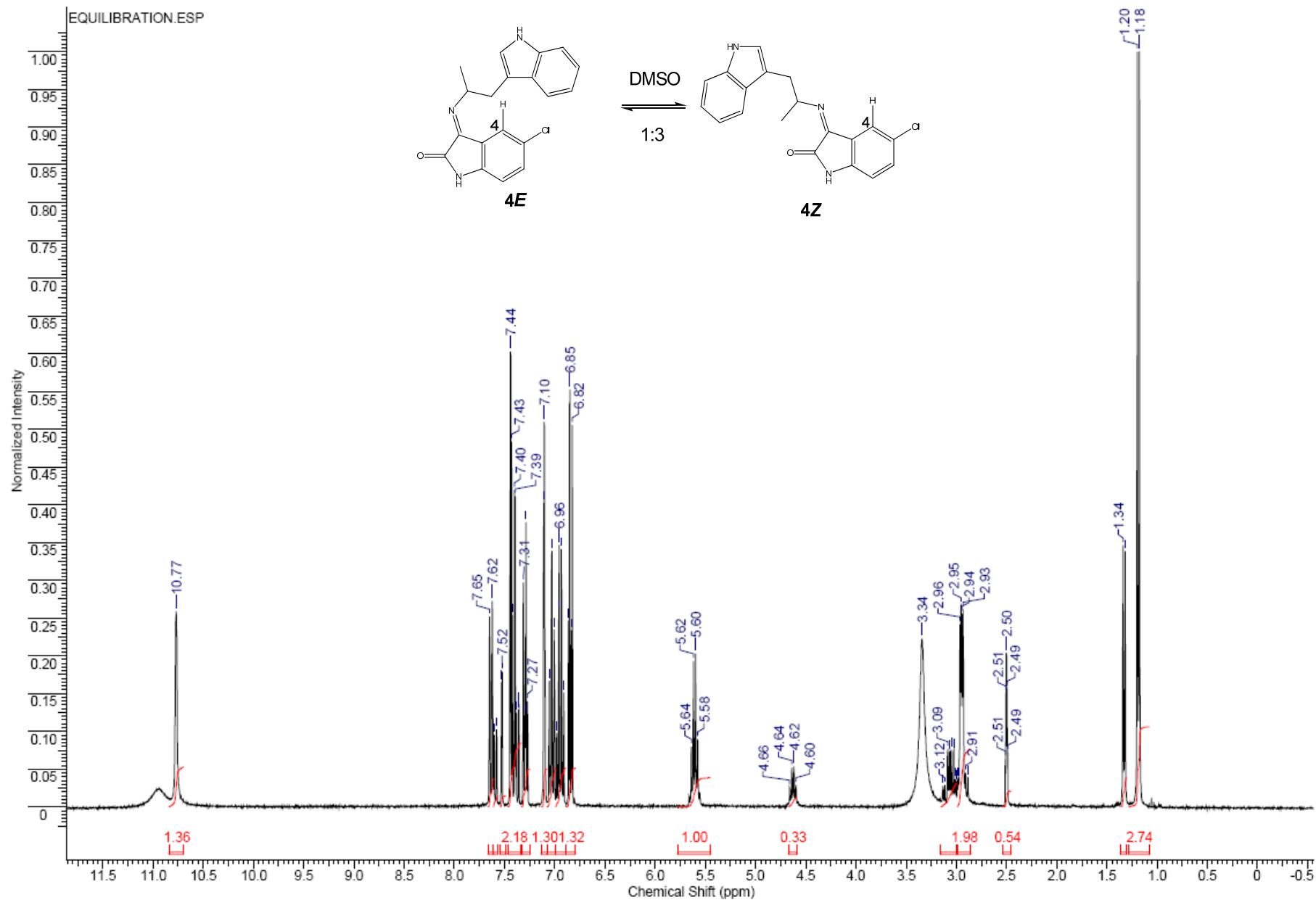

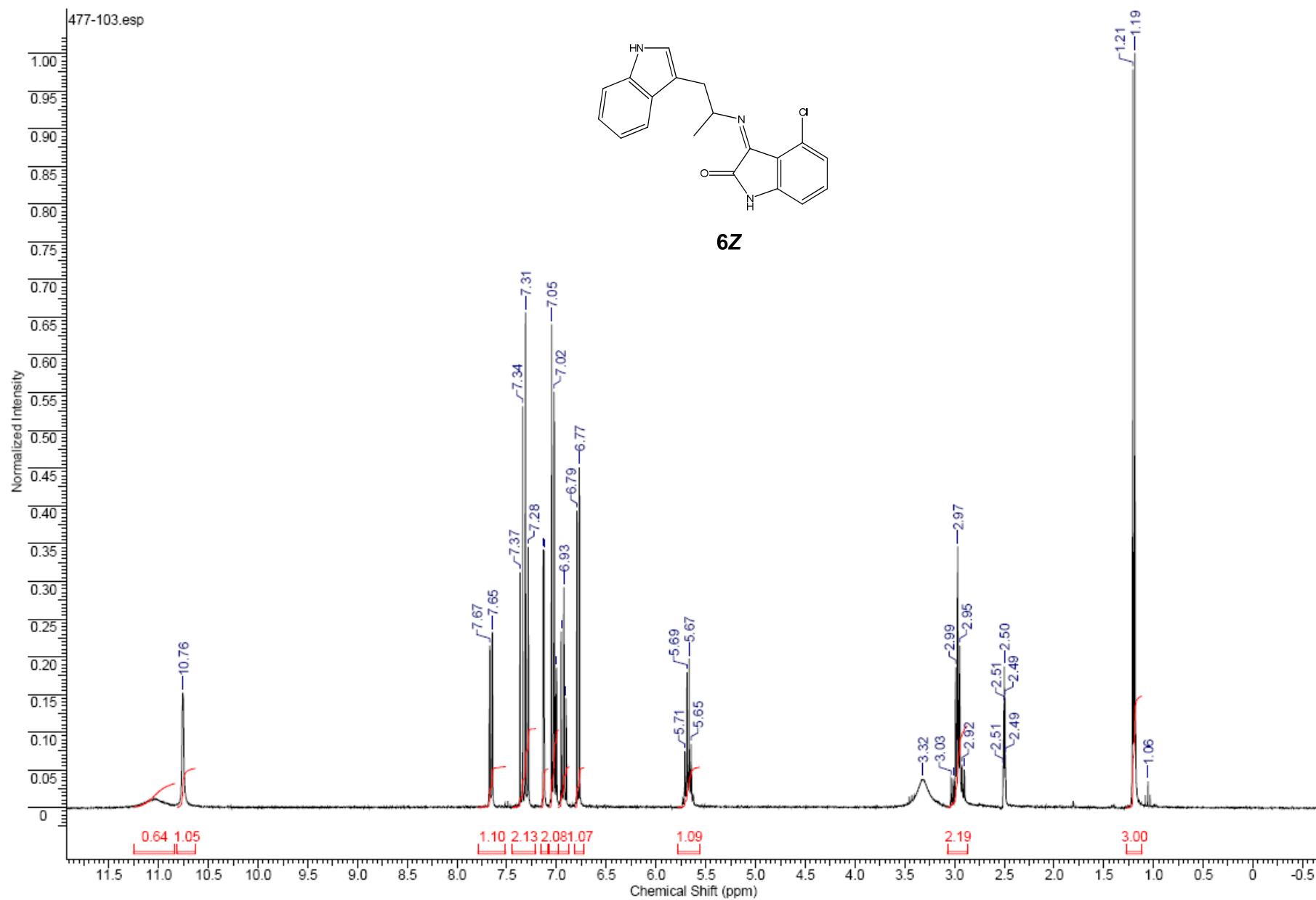

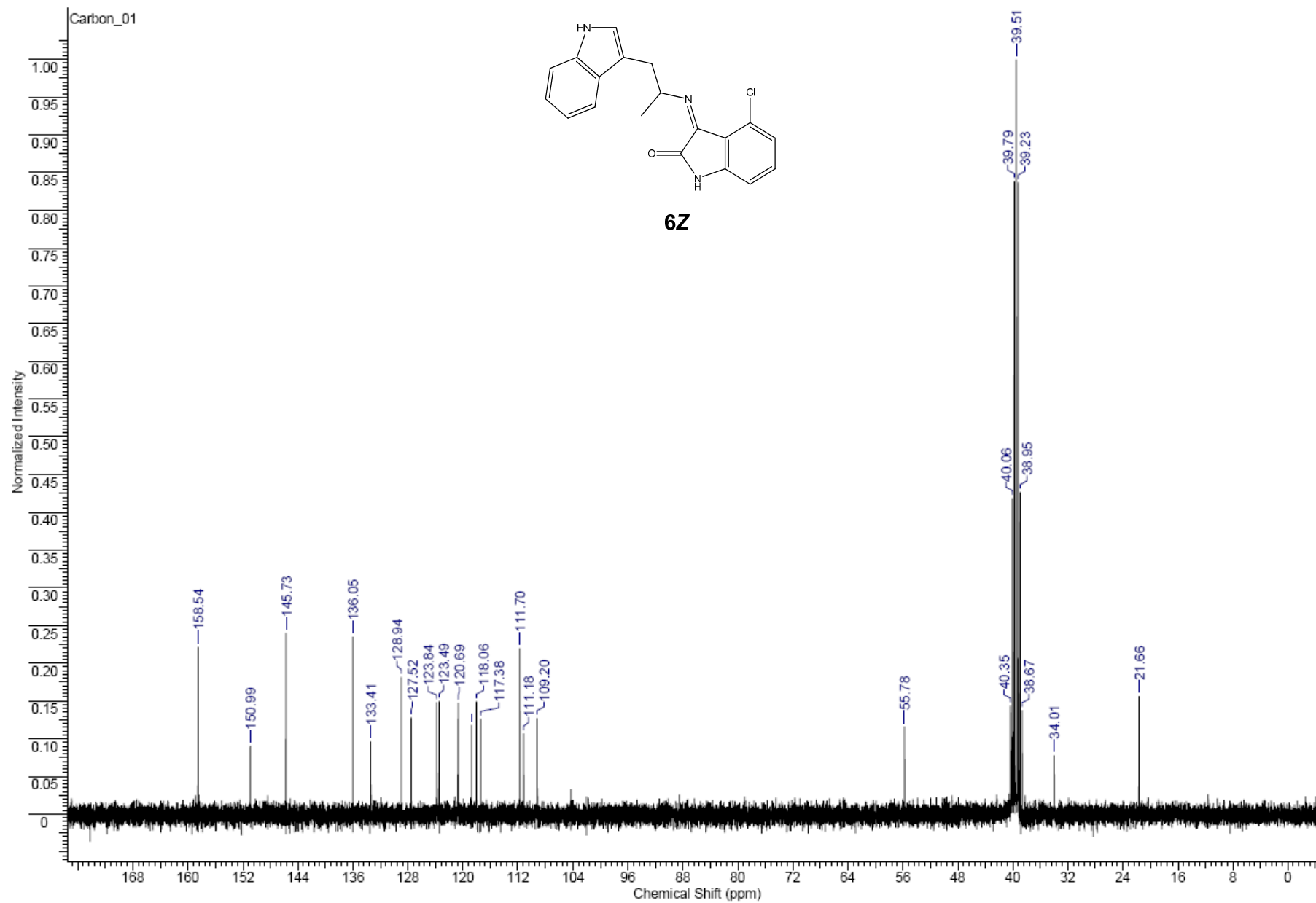

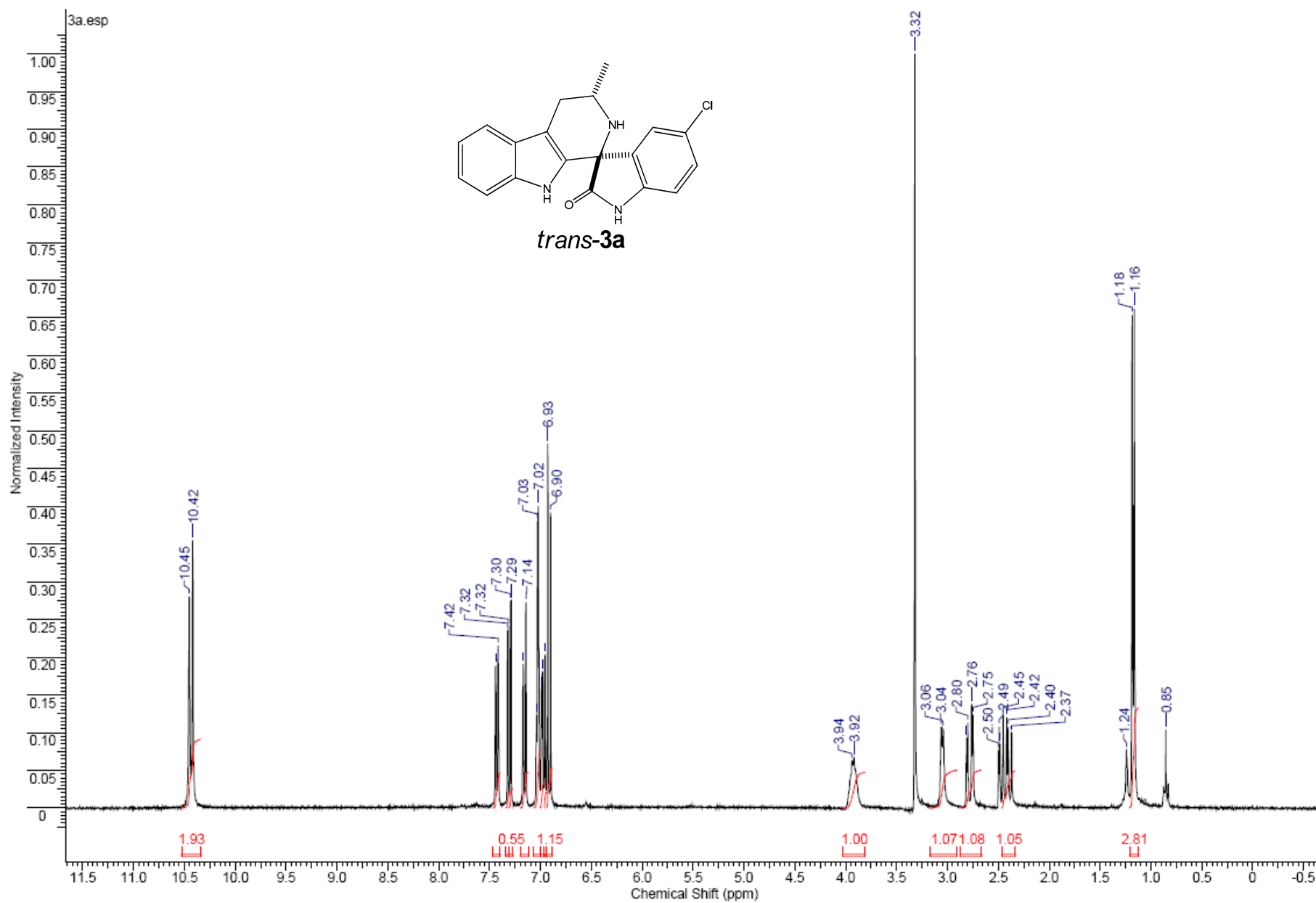

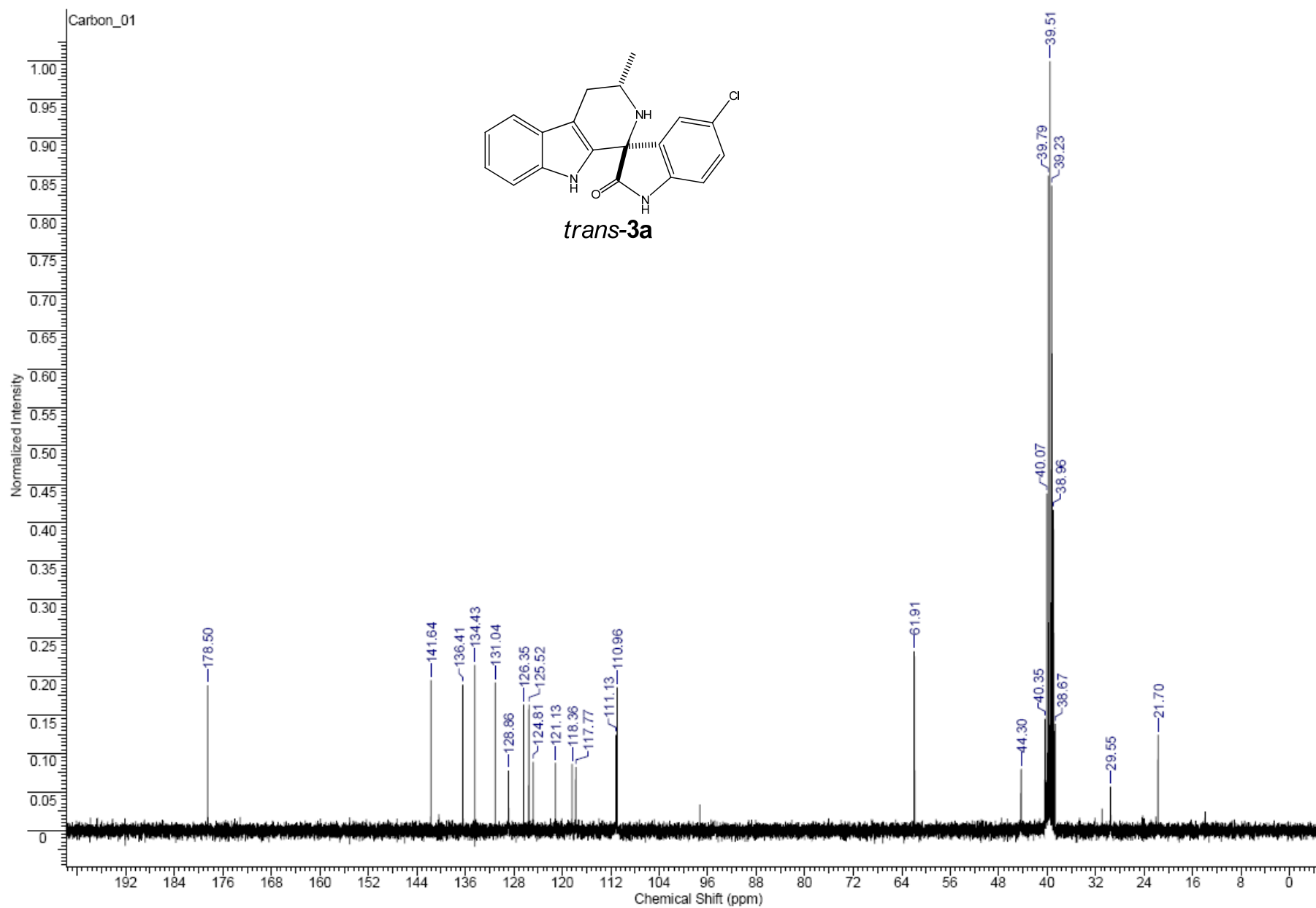

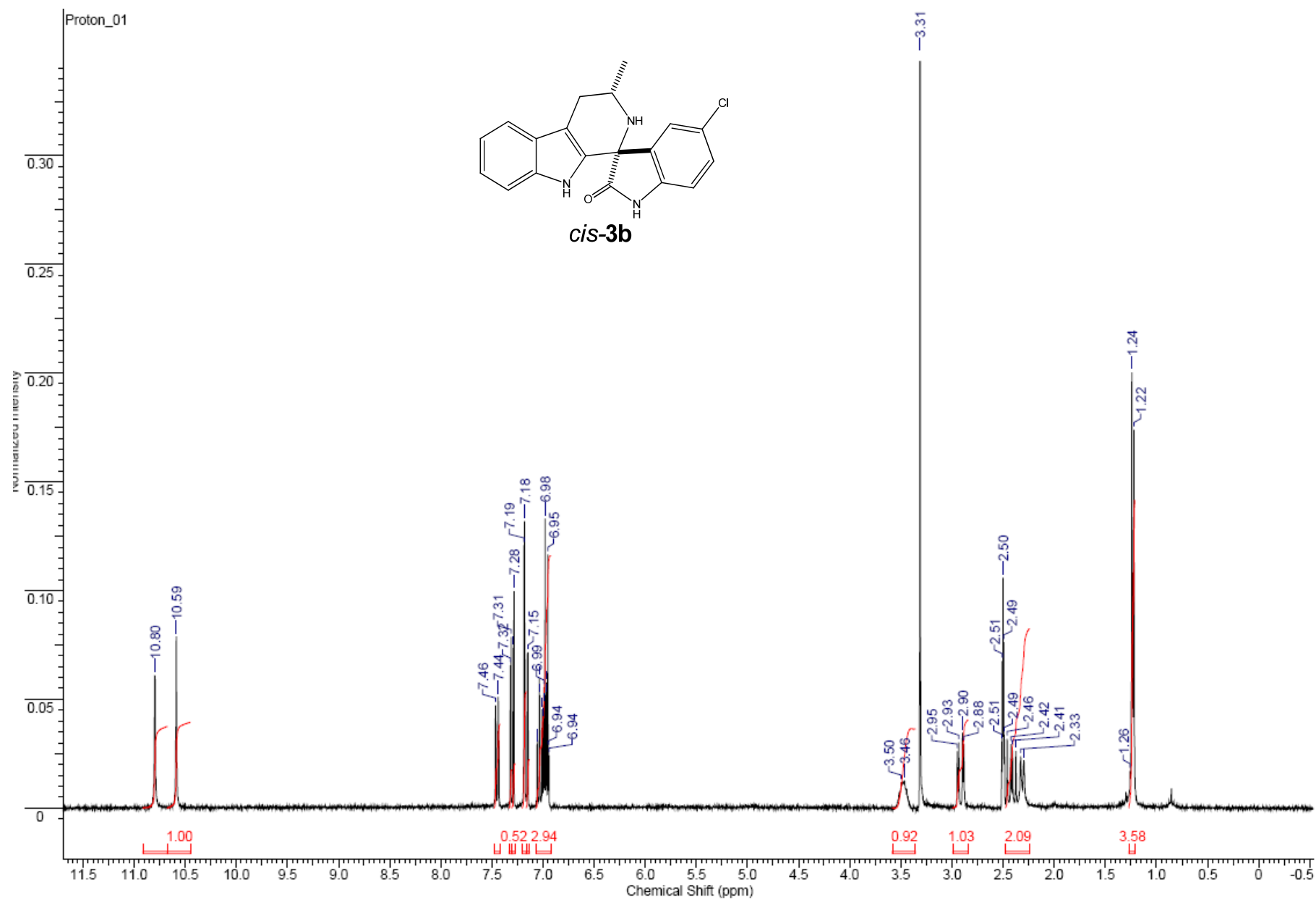

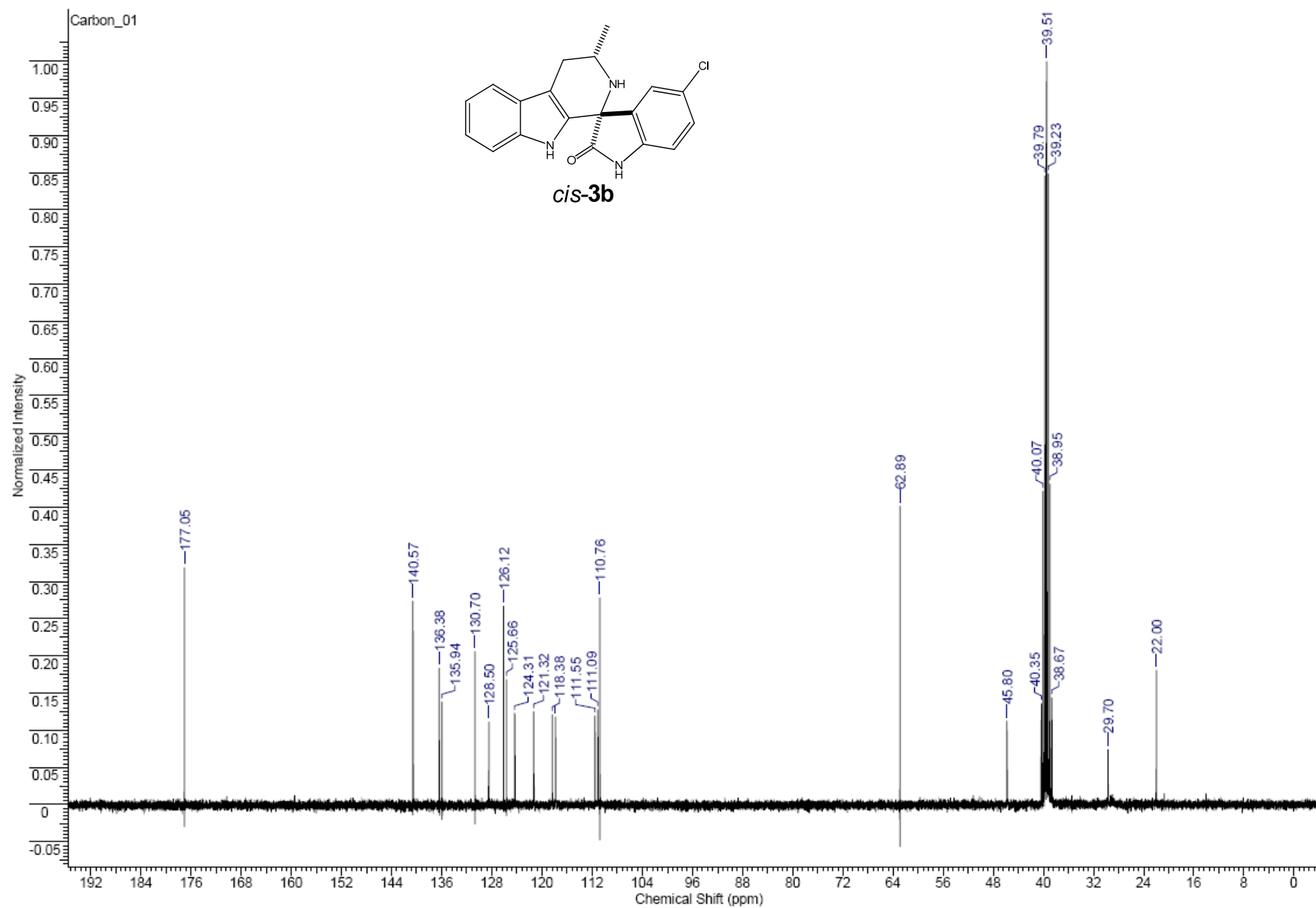

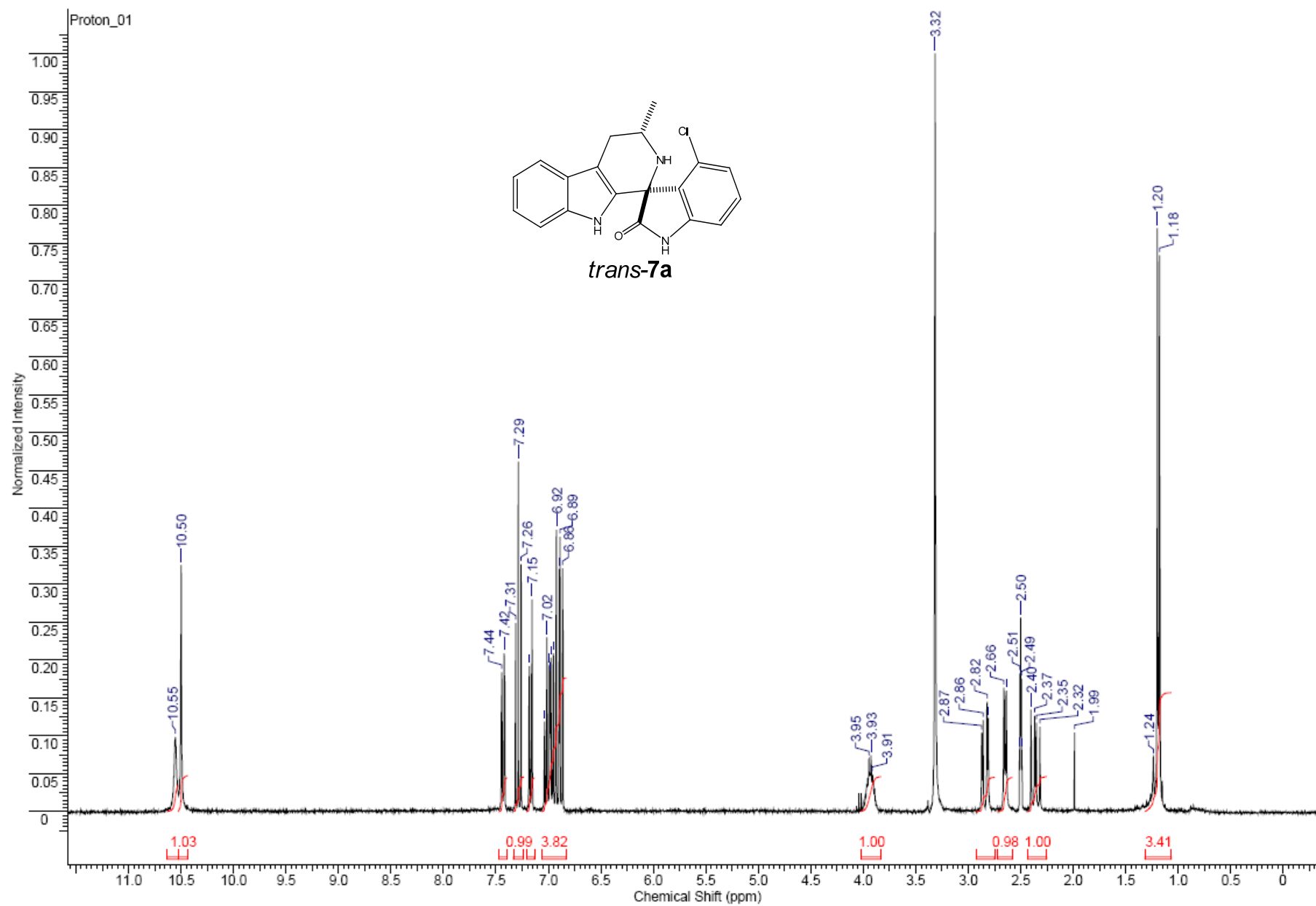

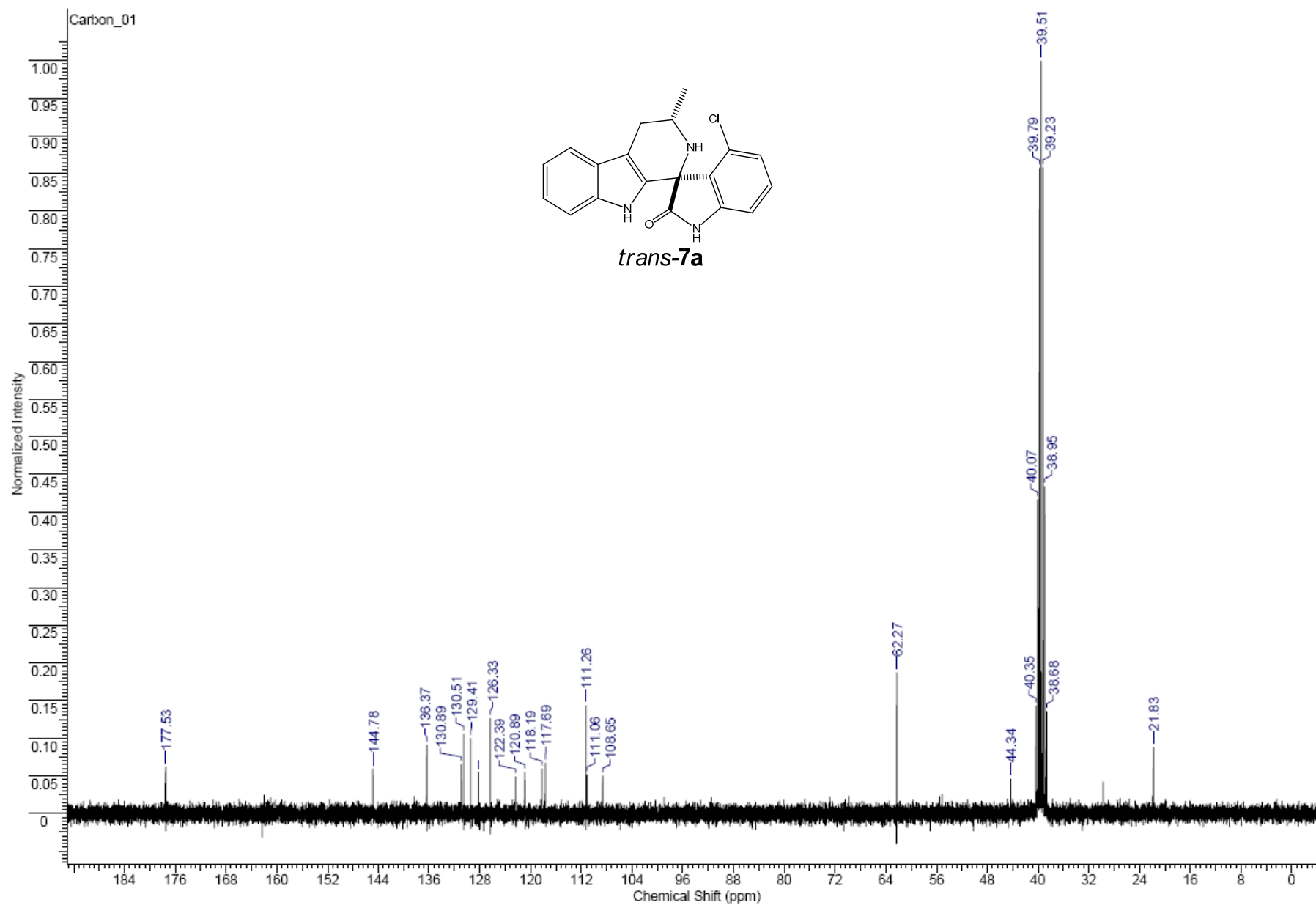

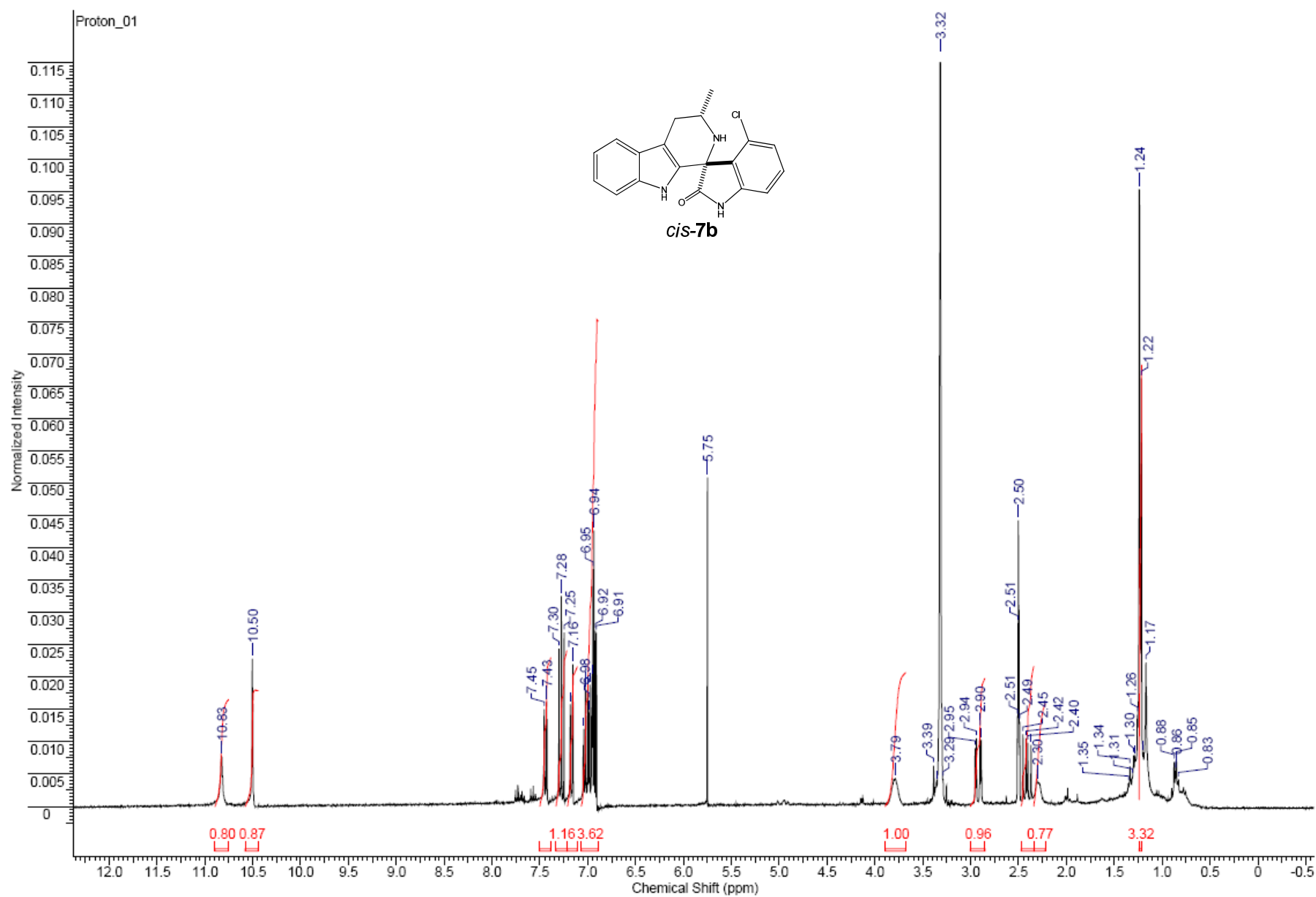

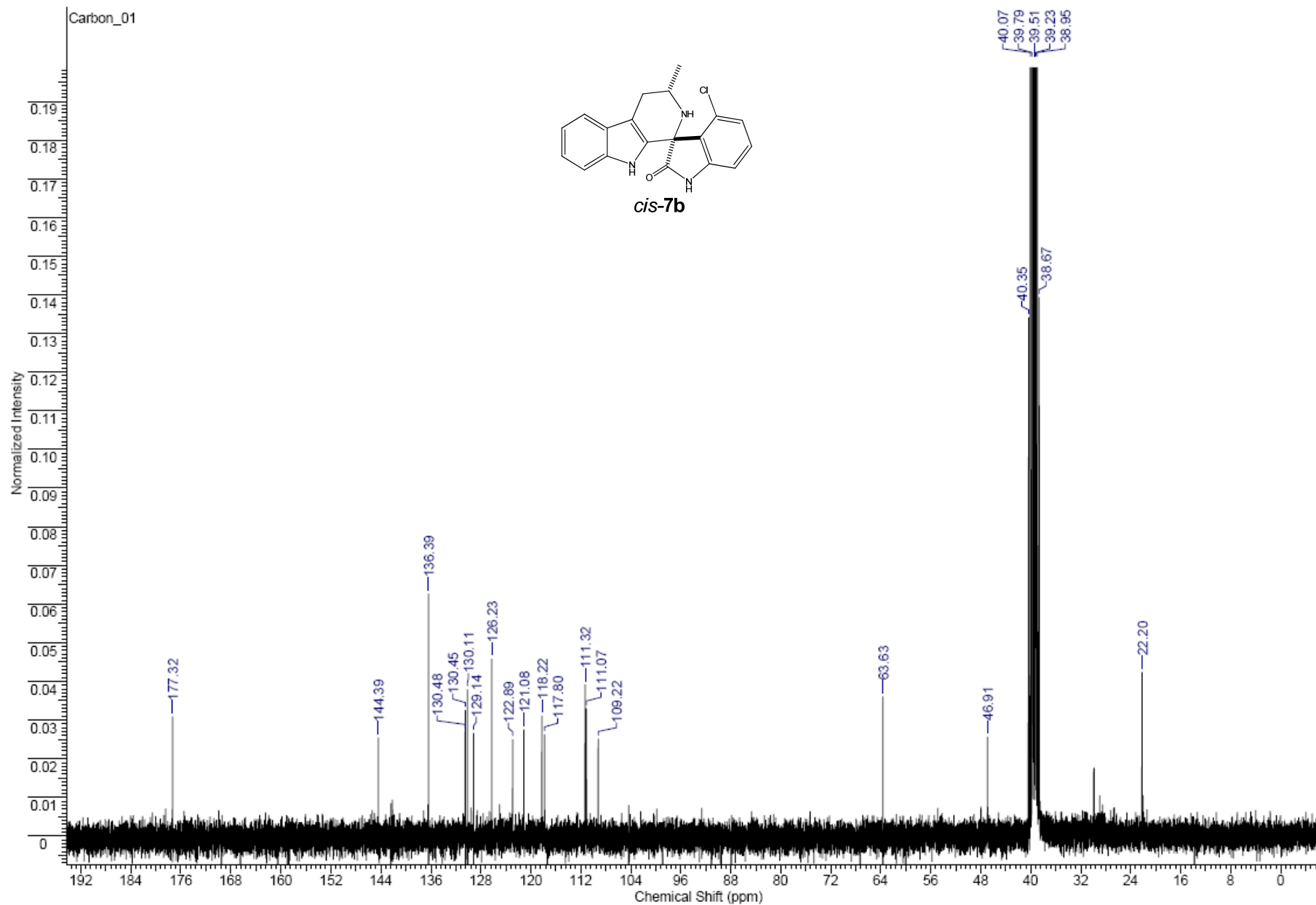

Supplement: Supplementary file 1 [file molecules-17-10131-s001.pdf]
